# First-person dimensions of mental agency in visual counting of moving objects

**DOI:** 10.1007/s10339-021-01020-x

**Published:** 2021-04-05

**Authors:** Johannes Wagemann, Jonas Raggatz

**Affiliations:** Institute for Waldorf Education, Inclusion and Interculturalism, Alanus University, Campus Mannheim, Am Exerzierplatz 21, 68167 Mannheim, Germany

**Keywords:** Quantification principles, Computer-metaphor, Mental action, First-person methodology, Focused attention/open monitoring, Consciousness-immanent model

## Abstract

Counting objects, especially moving ones, is an important capacity that has been intensively explored in experimental psychology and related disciplines. The common approach is to trace the three counting principles (estimating, subitizing, serial counting) back to functional constructs like the Approximate Number System and the Object Tracking System. While usually attempts are made to explain these competing models by computational processes at the neural level, their first-person dimensions have been hardly investigated so far. However, explanatory gaps in both psychological and philosophical terms may suggest a methodologically complementary approach that systematically incorporates introspective data. For example, the mental-action debate raises the question of whether mental activity plays only a marginal role in otherwise automatic cognitive processes or if it can be developed in such a way that it can count as genuine mental action. To address this question not only theoretically, we conducted an exploratory study with a moving-dots task and analyze the self-report data qualitatively and quantitatively on different levels. Building on this, a multi-layered, consciousness-immanent model of counting is presented, which integrates the various counting principles and concretizes mental agency as developing from pre-reflective to increasingly conscious mental activity.

## Introduction

Counting of moving objects is an elementary activity that not only relates to the structuring of our immediate perceptual environment but is also relevant for an abstract and operational understanding of various processes in society, science and technology. But what does it actually mean to count, for example, the ballet dancers performing on a stage or micro-organisms of a given species under the microscope? Early on in behavioral research three different quantification principles have been distinguished: subitizing, estimating, and serial counting (Klahr and Wallace 1973). Subitizing is the immediate and reliable determination of the cardinality of smaller sets, typically with a range of two to four items (Kaufman et al. 1949) which, however, can be slightly increased by canonical and temporally stable patterns like those of dices or dominos (Krajcsi et al. 2013). In contrast, estimating is understood as numerical representation of larger and more difficult to oversee quantities which is also relatively fast but leads to inaccurate results with ratio-dependent error (Gallistel 1990; Dehaene 1997; Xu and Spelke 2000). An alternative for larger set sizes is serial or one-by-one counting which costs considerably more time per item but leads to more precise results. While some researchers try to trace this more demanding process back to the other two forms of quantification (Meck and Church 1983; Gelman and Gallistel 1992), others suggest an independent model (Pylyshyn and Storm 1988; Kahneman et al. 1992). With the Approximate Number System (ANS) and the Object Tracking System (OTS), both approaches aim at hypothetical constructs which, on the one hand, should cover a wide range of phenomena and, on the other hand, should be traceable to specific neural mechanisms. The ANS is conceived to treat both continuous and discrete quantities in a continuous way and is therefore proposed to explain not only estimation but also subitizing (Dehaene and Cohen 1994) and serial counting (VanMarle 2015). The OTS, in contrast, is based on discrete representation of perceptual items to be counted by parallel as well as serial processes and hence serves for explaining both subitizing and one-by-one counting (Trick and Pylyshyn 1993, 1994).

However, this first exploration does not yet answer the question of what kind of activity counting essentially is. According to much literature the mentioned quantification principles and hypothetical models substantially rely on specific neural mechanisms (e.g., Feigenson et al. 2004; Mazza and Caramazza 2015). Nevertheless, earlier studies from the 1980s and 1990s were less concerned with neurophysiological evidence, but primarily with an approximative reproduction of subitizing, estimating, and serial counting by computer simulation (Church and Meck 1984; Sejnowski et al. 1988; Dehaene and Changeux 1993). Even though neural mechanisms correlating with various functional aspects of cognition have been found in the last decades, computer simulation still plays a major role in research. Especially in the context of counting and number knowledge, the information-processing view or computer-metaphor of the brain is ubiquitous—but it is also contestable regarding conflicting models, explanatory gaps, and the mostly neglected role of first-person experience and mental agency. From a reductionist point of view such differentiations of neural, computational, and mental activity may seem irrelevant, since the decisive processes take place on a physical level anyway. However, the computer-metaphor must be relativized by its classification in a historical series of other model conceptions of mind and brain, which were oriented to the respective technical achievements of an era (Daugman 1990). For it is speculative to conclude from the mastery of a technology to an ultimate understanding of the processes modeled by it. Why, in this respect, should the computational paradigm have a different fate from other anthropomorphizing models derived from ancient technologies (combustion, hydraulics, mechanics, steam engine) which have been established over the centuries and thrown over again in scientific revolutions (Kuhn 1970)? Besides more concrete reservations against the alleged similarity of brains and digital computers (Casey and Moran 1989; Ask and Reza 2016) it is above all the extinction of the experiencing and acting subject that can cast doubt on this comparison. While computers are technical devices designed by human minds for certain purposes, the same does not apply to the brain. The identification of brains or their parts with digital circuits would imply a mind-like user of this machinery in a dualistic way or attribute emerging mental powers to the biological “hardware.” These contradictory options directly lead to the hard problem of consciousness (the mind–body problem), which cannot be regarded as solved by third-person or materialistic approaches either (Chalmers 1995; Bennett and Hacker 2003; Wagemann 2011a; Majorek 2012).

A reason to include first-person data on mental activity in view of these problems is that certain cognitive processes, which are mostly considered to be restricted to automatic and subpersonal levels, allow, under appropriate conditions, experiential access and partly also control (Slagter et al. 2007; MacLean et al. 2010; Petitmengin et al. 2013; Wagemann 2020). In particular, for subitizing, traditionally considered a prime example of an automatic and pre-attentive process, there is evidence supporting a clear relevance of attention (e.g., Vetter et al. 2008), suggesting that even here the first-person perspective might provide new insights. A general difficulty for bottom-up computational accounts of counting is the reliable classification of “things” to be counted, especially when it comes to constitute sets of items that are highly heterogeneous (e.g., partly heard and seen, Kobayashi et al. 2004), temporally passing (e.g., jumps of a puppet, Wynn 1996), or non-physical (e.g., three wishes, Butterworth 2016). Here, generalization from incoherent, incomplete or ambiguous sensory input information seems hardly plausible, which rather suggests an involvement of attentional top-down processing (Kornmeier and Bach 2012; Wagemann 2020). Theoretically speaking, the apprehension of countable entities of any kind is a fundamental epistemological problem that cannot simply be reduced to pre-attentive bottom-up or computational mechanisms. A related point concerns the developmental acquisition of the logical basis of counting as expressed in the natural numbers system: “People cannot simply enumerate instances but must grasp, at least implicitly, general properties of the number system. Because the domain of ordinary physical objects and actions contains no counterpart to these principles, people cannot automatically transfer them from that domain” (Rips et al. 2008, p. 636/637). Hence, it could make sense to assume that humans have an innate grasp of “pre-existing concepts” to which number words are learned to map (Barner 2017, p. 565). While there already exist theoretical approaches which consider the relevance of attention and conceptuality for cognitive processes and, in particular, for counting (e.g., Baars 1988; Cowan 2001) this does not exclude to complement them by evidence originating from the first-person perspective. What can be expected from the latter are experience-based relations between specific forms of attention and conceptual coherence, which in principle evade the third-person perspective. This as well as an introspective investigation of the interaction of both aspects with sensory stimuli could provide important explanatory dimensions without stepping into the trap of psychologism (Kusch 2020) and will be discussed below in the context of Structure Phenomenology (Witzenmann 1983).

The questions, what kind of activity underlies cognitive processes, how they relate to conceptual content, and whether an agentive status may be ascribed to them, are also intensively discussed in the philosophical debate on mental action (Fiebich and Michael 2015; O’Brien and Soteriou 2009). If cognition essentially works on a passive, automated level, then mental activity plays at best an indirect and preparatory, if not epiphenomenal role for the whole process, which can hardly be considered as an intended action. However, if a more extensive conscious access to crucial aspects of cognitive processing is possible, then the mental activities involved would have to be understood as genuine mental actions. Roughly speaking, these options divide the debate into two camps one of which advocates a more restricted view (e.g., Strawson 2003; Mele 2009), while the other suggests wider and more fundamental accounts of mental action (e.g., O’Shaughnessy 2000; Peacocke 2007). Nevertheless, even if the former position often refers to an alleged dominance of unintentional automaticity (Bargh and Chartrand 1999)—which seems to converge with the neuro-centric and computational views mentioned above—it would be premature to identify automaticity as a general threat to mental agency. Rather, automatic processes can be conceived as subordinated partial performances of deliberately intended actions, provided that the actual intention is not undermined (Wu 2013). And furthermore, as already indicated, processes that have been automatized through learning and habitualization can be made at least partially conscious again and then also controlled to a certain extent. So it is not surprising that proponents of a more optimistic view of mental action in particular call for a methodological inclusion of introspection in philosophy (Soteriou 2013; Upton and Brent 2018), not to mention similar calls from the psychological side (Lieberman 1979; Locke 2009; Weger et al. 2019).

Despite such tendencies, it must be admitted that psychology and cognitive science still have some difficulty with introspection, at least from a methodological perspective. Although verbal reports already play an important role in some areas such as learning research and problem solving (Clement 2000; Goldin 2000), the rejection of introspection dominates among most scholars (Jäkel and Schreiber 2013). But just as the functional models of cognition are subject to epochal change, so too do research methods change over time and sometimes in unexpected ways. It should not be forgotten that psychology is a relatively young discipline that has detached itself from philosophy and, since its first consolidation in the twentieth century, has been oriented towards the empirical paradigm of the natural sciences. This reorientation of psychology was necessary to free it from the pre-modern influences of theological, scholastic and essentialist thinking. That in the course of this emancipation, however, at the same time introspection was discredited and rejected as a central access point to at least one of the two psychological core themes—behavior and experience—was not a necessity (Danziger 1980) and eventually led to “camouflaged introspection” in research procedures (Boring 1953, p. 169; see also Reisberg et al. 2003; Jack and Roepstorff 2003). Today, after the heyday of behaviorism and its cognitivist as well as constructivist readjustments, it seems overdue to take an explicit and offensive approach to the questions of a valid and reliable first-person access to cognitive phenomena and processes (Jäkel and Schreiber 2013). However, this approach must not ignore the historical achievements of psychology which should rather be included in such a way that an integrative paradigm can be envisioned. This needs to transfer proven quality criteria such as experimental replicability, independent test persons, and non-reactive data acquisition, in an appropriate way to empirical-introspective procedures without jeopardizing first-person phenomenality (Weger and Wagemann 2015a; Trnka and Smelik 2020). In order not to leave it here at a theoretical demand, the present study offers an exploratory study on the topic of visual counting combining third- and first-person methodology. In the following section, the experimental procedure is introduced with view on a moving-dots task (Sect. 2.1), data analysis is conducted according to a mixed qualitative-quantitative design (Sect. 2.2), and the results are presented (Sect. 2.3). In Sect. 3, partial models are developed for three analytical dimensions and condensed to a consciousness-immanent theory of counting which can also be understood as a generalized approach to cognitive problem solving. In Sect. 4, this work is critically reflected and discussed in methodological, psychological, and mainly philosophical regard. The conclusion (Sect. 5) provides a brief summary and an outlook for the future.

## Experimental procedure

### Stimulus, participants and task

**Fig. 1:**
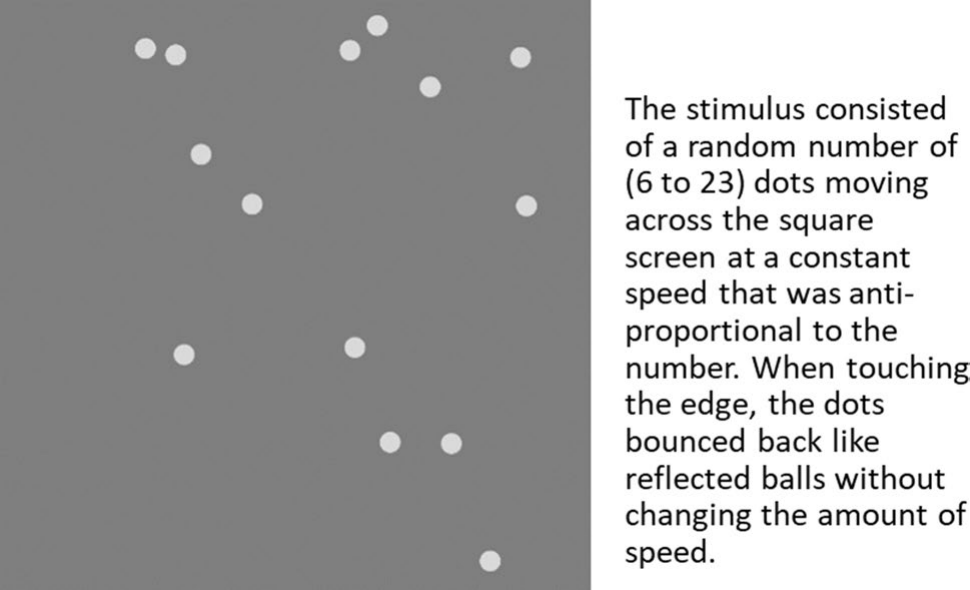
Snapshot of the stimulus

The stimulus used in this experiment consisted of a random number (between 6 and 23) of same-colored dots moving in various directions across a square screen at a constant speed that was anti-proportional to the actual number (programmed with Java Script, see Fig. 1). When approaching the edge of the screen, the dots bounced back like reflected balls, changing direction accordingly but not the amount of speed. Therefore, none of the dots disappeared, but they could temporarily overlap when they met. The stimulus was projected onto a screen in a seminar room at our university and presented for 1 min per trial to all participants simultaneously. At the end of each trial, the number of dots appeared on the screen. A few seconds after one trial, the next one was started with a new number of dots.

The test persons were 16 students in the third year of our Waldorf education BA-program (14 female, 2 male) with ages between 22 and 32 (*M* = 25.6) who participated in the context of a math course in which also psychological and qualitative aspects of numbers and numeracy were thematized—but of course no content related to our encoding categories, as explained in the next section. They were orally instructed to count as accurately as possible the number of the moving dots and to introspectively observe themselves how they would achieve this. They were also asked to pay attention to accompanying aspects of their thinking, feeling and perception. After the first three rounds, there was a 5-min break, during which the participants were supposed to take their first notes. At the end of another three rounds, i.e., after a total of six, they were asked to revise and supplement their protocols, which took them a maximum of 15 min. The form of data collection can thus be described as non-reactive, open-ended verbal self-report, the suitability of which has been explained elsewhere in particular for first-person access to fast cognitive processes such as perceptual reversals (Wagemann 2020).

### Data analysis

The protocol data were initially analyzed at a qualitative level using some features of grounded theory, albeit not in a canonical way (Glaser and Strauss 1967; Corbin and Strauss 1990). Firstly, in contrast to original grounded theory, the supposed inductive emergence of theoretical concepts from the data is seen critically according to social science (Dey 1999; Charmaz 2006; Kelle 2015) as well as science theory (Popper 1959; Fleck 1980). Apart from the general epistemological issue of abstractive conceptual derivation, alluded above with view on incomplete and ambiguous perceptual stimuli, data analysis is driven here by a clear research question based on defined psychological (e.g., estimation, subitizing, serial counting) and philosophical concepts (mental activity/action). However, this approach also differs from a strictly hypothetico-deductive attitude which typically aims at substantiating theoretical constructs with quantitative data without paying more attention to qualitative and first-person aspects. For, as described above, there is still no theory linking the psychological, philosophical and first-person aspects that could be directly empirically tested, but only initial ideas arising from the disciplinary literature as well as from our own preliminary investigations and earlier studies. We will show that despite this pre-conception of data analysis, there is still enough room for the peculiarities of the qualitative data and unexpected details that can contribute equally to a consistent coding scheme and a more concrete theory. Another point that deviates from common applications of grounded theory lies in the subject area of our research, which is clearly qualitative in nature but not accessible through external, object-related observation. Although originally developed for social science purposes, however, it seems possible to extend the notion of qualitative data to the internal field of privileged first-person data, as explained in the introduction. Moreover, it is precisely in this context that the thematically focused research question and the restriction of the data to task-related aspects appear as a safeguard against arbitrary, non-specific or excessive protocol data.

In this sense, data analysis circumvents open coding and orients to selective or focused coding under the core category of *first-person experience and mental activity/action in counting*—whereby the philosophical question about activity or action will be left to the discussion section. The core category also served as an overarching and falsifiable hypothesis for the experiment: We assumed that in this setting and with these instructions, the subjects are able to report via introspection on mental activities that they perform and possibly can control. If there was no indication of this in the data, we would have considered this as a refutation of the hypothesis. But since this was not the case, we distinguished three further dimensions of first-person experience and agency as subcategories for coding. While the first dimension refers to general features of the counting process such as invariant stages and moderating factors, the second delves into the subtleties of the central stage and differentiates it in terms of counting strategies and their variations. The third dimension again goes deeper into the second and describes the general mental dynamics that we have termed “focused attention” (FA) and “open monitoring” (OM), knowing that the reported phenomena do not correspond exactly to the use of these terms in the meditation literature. Overall, we can speak of a four-level hierarchical coding: (A) first-person experience and mental activity/action in counting (core category); (B) general process features; (C) counting strategies; (D) FA/OM-dynamics. While the core category has been motivated in the introduction, its sub-dimensions will now be differentiated into sub-categories and briefly introduced. For the *general process features* (B), we were initially inspired by a structure of mental activities that proved to explain the first-person data acquired in an earlier study with a task on directed thought (currently under review). On the one hand, this is obvious, since in both cases we are dealing with generalizable cognitive processes of problem-solving, but, on the other hand, it requires some modifications, since the current task includes sensory perception, which did not apply in the other case. After initial, only partially satisfactory coding attempts with the five directed-thought categories, we modified them and defined the following five categories accounting to the specific task and aspects found in the data:

1. *Counting* This category refers to all verbal expressions concerning specific strategies of counting and therefore the counting process in the narrower sense. Variations and combinations of strategies are also included.
2. *Motivation* This includes all statements about the individual motivation and concentration of the participants and how it changes over time, for example that engagement seems strenuous or easy or that one gets tired after some rounds.
3. *Positive evaluation* Here, verbal expressions are relevant indicating that the participant experienced certainty or success, be it after comparison with the official counting result or before and independent of it. Expressions of positive feelings are also included.
4. *Negative evaluation* This category is reverse to category 3, the participant experiences uncertainty before or during an attempt or failure afterwards. Reasons for this can also be given as well as negative emotions expressed.
5. *Checking* This category includes all statements expressing that participants reconsider and cross-check their first result with a second count. This can also be mentioned indirectly, for example if a second result differs from the first count.

For the dimension of *counting strategies* (C), subordinated to Category B1, we aligned the coding categories with the quantification principles explained in the introduction, but also added—as indicated by the data—a further category which is not mentioned in the counting—literature to our knowledge. Although, in contrast to the first-person character of the data, the original definitions of the counting principles rely on external behavioral measures (e.g., reaction time, accuracy), we decided to use the same designations for the categories to avoid unnecessarily complicating the terminology. Nevertheless, it will turn out that most of the pivotal aspects can also be proven with introspective data, even if only qualitatively or indirectly.

1. *Estimating* Without an exact counting technique, an attempt is made to grasp the total number of objects at a glance.
2. *Subitizing* Smaller quantities are accurately recorded at a glance (typically 2 to 4). Advantageous are spatial proximity, same direction of movement or canonical (e.g., dice or domino) patterns. For quantities of 5 or more, this technique is still partly possible, but it depends increasingly on whether the objects appear in canonical patterns.
3. *Serial counting* All elements of a set are individually grasped and counted one by one. The goal is (as with subitizing) an exact result, but the process requires more time.
4. *Partitioning* The perceptual field is divided into search sections (e.g., up/down, right/left, quadrants). For the individual search sections, (different) counting strategies (categories C1–C3) can then be applied. Alternatively, different directions of the spatial search movement are expressed (vertical/horizontal/diagonal).

While the first three categories have been already explained above, the last one must be clarified now. By studying the verbal reports, we came across the aspect of partitioning meaning that the perceptual field is subdivided into sections (e.g., left/right, above/below, four quadrants) which are processed successively or that processing (particularly serial counting) is conducted in certain spatial directions (e.g., in horizontal rows, vertical columns, diagonal). Because partitioning as such does not comprise counting, it can be considered an auxiliary tool which is combined with one of the other three principles.

The analytical dimension of FA/OM-dynamics (D) further refines and at the same time generalizes typical forms of mental activity observed in the individual counting processes. This takes especially account of the first-person characteristics of a process involving sensory perception, insofar as it relates to the dynamic structure introduced in two studies on voluntary perceptual reversals (Wagemann et al. 2018; Wagemann 2020). Modifications are necessary here too, however, because although counting seems to be associated with a perceptual reversal due to changed conceptual patterns (the numbers), ever new (increasing) numbers must always be ascribed to the stimulus. Therefore, also in view of the data that did not show finer differences, we decided to group the earlier four categories to two more general types of mental activity which can also be associated with focused attention and open monitoring as known from quantitative and qualitative meditation research studies (e.g., Lutz et al. 2008; West 2016; Wagemann 2011b). Here, we consider FA as a mental micro-gesture that turns attention towards the stimulus and individualizes a conceptual pattern to a single case, and OM as a mental micro-gesture that turns away from the stimulus and extends to wholeness. Note, however, that this understanding of FA and OM extends current definitions in that it is not limited to purely attentional modes, but includes references to various (individualized, holistic) forms of mental content. In particular, we see OM not only as a form of awareness detached from specific content, but also as the ability of controlling holistic aspects of cognitive processes. Moreover, in the context of problem solving, this can also refer to convergent and divergent forms of thinking, as will be explained in the section on theory building. This analytical dimension is more explorative and therefore not further differentiated in sub-categories, it summarizes aspects of FA- and OM-like forms of mental activity in this sense, and their dynamic interchange.

In addition to the qualitative analysis, the coded data were analyzed quantitatively in part. Methodologically speaking, this corresponds to a “sequential qualitative-quantitative design” (Kelle 2015, p. 595; see also Creswell and Creswell 2018; Glaser 2008). Quantitative analysis was restricted to categories (B) and (C) and started with an evaluation of intercoder reliability between the independent codings, and then, an analysis of the percentage shares of the coded data in the categories was conducted, as will be shown in the next section.

### Results

The protocols were relatively short, containing between 87 and 420 words (237 on average), and were formulated in bullet points with more or less complete sentences. In order to convey an impression of the verbal data and the variety of descriptions, exemplary excerpts are given in Table 1. Before outlining the coding procedure from which this compilation results some general aspects and individual formulations shall be highlighted. At first it was noticeable how focused and largely without digressions in content the protocols were written. It was also interesting to see how individual the verbal expressions were, although they were related to a limited range of phenomena. And it became clear that the test persons approached the task from sometimes quite different perspectives; in the end they worked with the same quantification principles, but with individual weightings and combinations. This shall now be illustrated with some exemplary formulations: “I counted very fast in the second round and was able to distinguish the counted from the uncounted points for a moment, despite the movement” (Part. 8). “I started counting in a quarter of the square, but at the same time strained my eyes to see the whole square and the movements of the balls … internal differentiation of the individual balls, although they look the same” (Part. 10). These two excerpts point to the challenges of keeping track of and distinguishing the moving objects into already counted and uncounted items. The participants also express how they cope with the challenge: While Participant 8 emphasizes her high-speed counting Participant 10 mentions aspects of her spatial strategy, both of which can contribute to succeed in the task. Another interesting aspect becomes apparent in the following: “Lips accompany counting” (Part. 9). “While counting, I observed myself that I would have liked to count out loud or write down intermediate results, but that would probably have been disturbing” (Part. 14). This shows the inclination to support counting by bodily means or actions, although that seems to be something that can also be suppressed.

**Table 1:** General process features: exemplary excerpts from the self-reports

Cat	B1	B2	B3	B4	B5
Part	Counting	Motivation	Positive Evaluation	Negative Evaluation	Checking
1	Counting dots in groups/pairs; the black field divided (in imagination) into four parts, dots counted by field and added afterwards	–	–	This method is not always successful due to the speed	By estimating and then counting to confirm
2	They were divided into two groups at the top and bottom of the screen, they were sorted and I could count them more easily	For me it was motivating for the next sequence if I had the right number of points in the sequence before	On the other, hand I always had the right result for the few points	They overlapped much more often and I did not know whether I had already counted them or not	and then I counted them all again
3	I then divided into groups of three and looked, where is the rest?	The longer you do this exercise, the harder/more strenuous it becomes	I was surprised that I counted correctly in the first two rounds	The fourth time I couldn't even come up with a number, I was kind of out	–
4	I try to see number-groups and grasp connections	Meanwhile ups and downs (giving up despondently, new picking up, motivation)	And was (probably rather accidentally) right	–	–
5	Try to always intercept “pairs of points” and freeze the image internally at this moment	Stress level at round 1 high, … increases with increasing number of points and duration of the exercise	–	Search for a foothold and a starting point	–
6	By looking “inaccurately” (rather at the black screen) it is easier to count	At first, counting was fun, because it is structured like a game and it stimulates the own ambition to count correctly	When the result was announced, “I was close enough to it” (to the actual 18 points) and I was happy	–	After the first count this certain number had quickly “fixed” in me and although I tried to recount several times, it did not change
7	Mostly I count 2, 4, 6, 8 or 3, 6, 9 → that means I look when balls meet and count them together	If there are a lot of bullets, you are more likely to lose the motivation to keep counting, because you get mixed up faster	You're glad when you find out the “right” number…	… disappointed when you count "wrong". … if you lost one in the field, you had to start all over again	–
8	I also divided the area into fields and tried to concentrate on one, took a "photo" again and tried to put it in relation to the other fields (this was rather estimated then, but easier with the many points)	–	That went well with fewer and faster points, too … overall, I felt safer in the second round … and trusted my result more	–	If I subsequently deviate from the first census/estimate
9	Count quickly and accurately by waiting for a “suitable” position of the balls, e.g. when many are in a corner	Do not lose patience and stay concentrated or focused	–	… and the results are becoming increasingly inaccurate	–
10	… looking for orientation at the edge of the square, starting to count in a quarter of the square, but at the same time straining the eyes to have the whole square and the movements of the balls in view	Concentration decreased after the 3rd run, it became more strenuous to focus the eyes	I was sure that I was right	–	When I counted 2–3 times and checked my result
11	I start counting at the top left or top right and then go further down, trying to capture the whole field and the other points and their movement from the corners of my eyes, counting in groups of 2 to 4, capturing the groups and adding them up	Alertness and concentration necessary for counting: inner activity and effort after several passes makes counting exhaustive	Yet I was able to count them all correctly	–	–
12	I always started at the bottom edge, when some balls bounced, then my eyes went up quickly. I collected several balls at once and added them to the sum I had calculated up to that point	From the very beginning I was tense, expectant, excited and highly concentrated	If I recorded the correct number, it usually happened right at the beginning	From 20 or 21 bullets on there were too many. The best I could do was guess	–
13	Optically tried to count, always tried to form pairs of two balls	Emotional detachment from right and wrong counting, i.e. not allowing too much joy or too many negative feelings about the final result	Observation: 2 of 3 correct	But which have blended too quickly	–
14	After that I decided to watch a corner and wait for a moment when as many points as possible were there and they were easy to count, then I quickly memorized the points in the corner and counted the points in the rest of the field	When the first picture was shown, I was already a little overwhelmed, in the sense that I thought it might be difficult to count the dots	To count the dots correctly triggers a certain feeling of happiness	I was initially surprised by the mass of dots and did not know exactly where to start counting	To be on the safe side I wanted to count again (with the same method)
15	At the very beginning of the first count I took all the balls into my consciousness, I focused on single balls and counted very quickly	–	But I knew that the first result was the most reliable, which was correct	With the faster and less points I could not get this “distance” … I always arrived at different numbers	when I then had a number, I tried to check my first result by systematic counting
16	First I took a close look at the balls. As the balls moved faster and always came together in groups, I started to count them. If they moved in a jumble, I concentrated on the lower area and counted them, then I started to count the upper area	I couldn’t check it anymore because my concentration was lacking at the end	So, I came up with 18 balls	Because I had the impression that the balls were moving so fast that I couldn't even count them. I lost track	I then counted the balls one by one for control

These 61 text-fragments resulted from coding according to the dimension of *general process features* (B) with its five subcategories. These excerpts are exemplary as they do not necessarily display all data from the individual protocols falling under the respective category

Regarding the categorical schemes, exciting discoveries could also be made. Against the background of general process features (B), the following text-example is difficult to assign unambiguously to one of the categories: “Often I stayed with my concentration at one point of the field to count, then my eyes briefly jumped to the whole field and then back to that point.” (Part. 7). On the one hand, this might be associated with Category B1 in the sense of a spatial auxiliary technique or, on the other hand, it could be interpreted as motivational attention regulation (Category B2). There are other examples of text-fragments that can rather be localized between two categories than exactly in one: “When I was finished, ‘a switch was flipped’ and I was no longer concerned with the points, but rather with my thoughts and weighed the correctness of my result for myself” (Part. 8). This seems to reflect an intermediate stage between counting (Cat. B1) and evaluation since one has finished counting but not yet arrived at a positive (Cat. B3) or negative assessment (Cat. B4). Apart from such special cases, a predominant number of text-fragments could be reliably coded according to the five categories. One of the two authors (JR) fragmented 63 text records out of the data according to the five categories, this was checked by the other author (JW) in a second, independent coding of the fragments. Intercoder reliability according to Cohen’s Kappa yielded *κ* = 0.939 which is almost perfect or even excellent agreement (McHugh 2012; Dawson and Trapp 2004). Only three out of the 63 assignments deviated; two of these fragments were excluded from coding since they allowed interpretation beyond the strict categorical scheme (as the above example of Part. 8). The third discrepancy could be clarified, so that this text-fragment could be consistently classified within the categorical scheme. So, in the end, we had 61 text-fragments encoded with full agreement (as displayed in Table 1); the further quantitative analysis only refers to those.

Quantitative analysis of the general process features includes the relative frequencies of coded text-fragments over the test persons (Table 2). As to be expected according to the instructions, all participants made statements about their counting strategies, while the aspects attributable to the other categories were lower, although not below 50%. Before further interpretation of the results, we regard this as a positive indication that the categories are suitable to represent valid aspects of first-person experience and activity. At least the full coverage of Category 1 can be taken as justification for a more fine-grained sub-coding according to the counting strategies (C) just at this point. Not surprisingly, many verbal expressions about the three quantification principles discussed above could be found in the data (Table 3). However, quite a few statements about spatial partitioning (Cat. C4) were discovered as another principle which, as mentioned, has not been dealt with in the context of counting so far. This may be because partitioning is not an independent counting strategy, but only makes sense in combination with one of the other three principles. Nevertheless, partitioning plays an independent role in our study in so far as it contributes to the diversification of individually selectable counting strategies, as will be shown more precisely. For the 46 text-fragments coded according to the C-categories, intercoder reliability yielded *κ* = 0.794 which is lower than the κ-value for the B-categories but can still be seen as a fairly good (substantial) agreement. Remarkably, in 4 out of 7 cases where the two coders disagreed the second coder (JR) noticed ambiguities and indicated the category coded by the first coder (JW) as an alternative option. One of these examples reads as follows: “To count as many points as possible when clearly arranged” (Part. 14). With view on “as many points as possible” which can be associated with an open-ended serial counting this has been assigned to Category C3 by the first coder. When emphasizing “clearly arranged,” as by the second coder, this piece of data could also be seen as an instance of Category C2 (Subitizing). Apart from such ambivalent cases, the most part of the data could be reliably coded. In retrospect, as obvious from the quantitative analysis (see Table 4), the inclusion of partitioning in the categories is confirmed by its frequency in the protocols (81.3%), only exceeded by subitizing (87.5%). To confirm that most subjects used several, typically three counting strategies, we refer to Table 5.

**Table 2:** General process dynamics: percentage distribution over test persons (*N* = 16)

Category	B1	B2	B3	B4	B5
	Counting	Motivation	Positive evaluation	Negative evaluation	Checking
Coded text fragments over test persons	100%	81.3%	81.3%	68.8%	50.0%

**Table 3:** Counting strategies: exemplary excerpts from the self-reports

Cat	C1	C2	C3	C4
Part	Estimating	Subitizing	Serial Counting	Partitioning
1	By guessing…	Counting dots in groups/pairs	… and count afterwards	The black field divided (in imagination) into four parts, dots counted by field and added afterwards
2	First roughly counted	–	Recounted accurately	They were divided into two groups at the top and bottom of the screen, they were sorted and I could count them more easily
3	–	I then divided into groups of three and looked, where is the rest?	Tried to count by quick eye movements	Always look in a corner and count how many bullets appear
4	With many points I begin to estimate	I try to see number-groups and grasp connections	Track and count each point on its way individually	–
5	–	Try to always intercept "pairs of points" and freeze the image internally at this moment	Count fewer points	I often start at the bottom and follow all the points on the way up or I look down but stay in the following rather than the expecting attitude
6	By looking “inaccurately” (rather at the black screen) it is easier to count	–	–	–
7	–	Mostly I count 2, 4, 6, 8 or 3, 6, 9 → that means I look when balls meet and count them together	Count individual balls 1, 2, 3, 4, 5	Count the number of balls that hit one side of the square
8	Getting a feeling for the rhythm of the balls in order to estimate their quantity	… until the points have come together to be able to count many at a glance	–	Divided the area into fields and tried to concentrate on one
9	–	Count in steps of two or three	Count quickly and accurately …	… by waiting for a “suitable” position of the balls, e.g. when many are in a corner
10	–	Have the whole square and the movements of the balls in view	–	… looking for orientation at the edge of the square, starting to count in a quarter of the square
11	–	Counting in groups of 2 to 4, capturing the groups and adding them up	Few, fast points: counting is easier because you can follow the movement of the other points in parallel	I start counting at the top left or top right and then go further down
12	I could only guess at best	I collected several balls at once and added them to the sum I had calculated up to that point … dice patterns	–	I always started at the bottom edge, when some balls bounced, then my eyes went up quickly
13	Often estimated if it looked like too many balls	Optically tried to count, always tried to form pairs of two balls … to recognize patterns	Tried to count fast	Background divided into areas mentally into smaller fields e.g. top right and left, bottom and top etc
14	–	I tried to perceive the picture as a whole, to stare at it, and thus to capture all points	To count as many points as possible when clearly arranged	Watch a corner and wait for a moment when as many points as possible were there …
15	Counted by feeling	Wait for “favourable” moments in the patterns	Focused on single balls and counted very quickly	–
16	–	Here I proceeded not in pairs of two, but in groups of three	In this round I counted the balls one by one	I concentrated on the lower area and counted them, then I started to count the upper area

These 46 text-fragments resulted from coding according to the dimension of *counting strategies* (C) with its four subcategories. These excerpts are exemplary as they do not necessarily display all data from the individual protocols falling under the respective category

**Table 4:** Counting strategies: percentage distribution over testpersons (*N* = 16)

Category	C1	C2	C3	C4
	Estimating	Subitizing	Serial counting	Partitioning
Coded text fragments over test persons	50.0%	87.5%	75.0%	81.3%

**Table 5:** Number of strategies used by testpersons

Number of strategies used	1	2	3	4
Number of test persons	1	1	12	2

For the last analytical dimension of FA/OM-dynamics (D-Categories), we found evidence in the protocols of 10 out of 16 participants and indicated references to FA and OM in square brackets (see Table 6). On closer inspection, even though we refrained from sub-categorization at a formal level, different aspects of FA and OM could be distinguished in the data. Focused attention, on the one hand, comprises a more dynamic intentionality or orientation to individual parts or aspects of the stimulus (“Concentrate and look closely”, Part. 4; “focusing precisely”, Part. 10); on the other hand, it passes over in static states of fixing specific situations accessible to memory (“Freezing the image internally”, Part. 5; “take a ‘photo’ of what I see to stop the movement”, Part. 8). A similar distinction can be applied to open monitoring: At two levels mental gestures of distancing and detaching from singular aspects occur the first of which still include task-related references in a holistic sense (“see the whole square and the movements of the balls”; “keeping the overview and distancing oneself again”, Part. 10), while others drop engagement in the task in order to have a short recovery break (“Avert my eyes from the screen”, Part. 9; “I looked out of the window to refocus”, Part. 14). Before these subtleties are examined in more detail in the next section, it may suffice here to state that these mental activities occur in the context of the counting process, but independently of certain counting strategies. This is most likely to be related to the auxiliary technique of partitioning, which also oscillates between individual and holistic aspects of the stimulus.

**Table 6:** FA/OM-dynamics: coded data

Part	(D) Focused attention/open monitoring dynamics
4	Concentrate and look closely [FA]
5	Freezing the image internally in this moment [FA] … Alternation between short breaks in concentration [OM] and the will to count [FA]
6	I also noticed that it is of little use to concentrate only on one point [FA], but it can be counted more easily by looking “inaccurately” (rather at the black surface) [OM]
7	Often, I stayed with my concentration at one point of the field to count [FA], then my eyes briefly jumped to the whole field [OM] and then back to that point [FA]
8	First, I tried to take a “photo” of what I see to stop the movement [FA]
9	Avert my eyes from the screen again and again for brief moments [OM]
10	Started counting in a quarter of the square [FA], but at the same time strained my eyes to see the whole square and the movements of the balls [OM] … Mixture of thinking into something, focusing precisely [FA], keeping the overview and distancing oneself again [OM]
11	I try to capture the whole field and the other points and their movement from the corners of my eyes [OM]
14	Afterwards I looked out of the window [OM] to refocus [FA]
15	With the faster and less points I could not get this “distance” [OM failed]

In the square brackets, an interpretation of the data as focused attention (FA) or open monitoring (OM) is indicated

## Theory building

In this study, as became clear, quantitative analysis has a quite different significance compared to mainstream behavioral studies. For it is not about validating theoretical constructs or neural mechanisms in a formal way or simulating computational models but rather building a theory of counting based on qualitative first-person data and aiming at differentiated forms of mental activity. Therefore, quantitative analysis here serves more as an informal indicator in the course of qualitative analysis than as a goal in itself. Thus, the roles of both types of analysis are reversed and the first-person aspects, which are otherwise often neglected, come to the fore. To keep the focus of this first exploratory study, we have also refrained from investigating other variables, such as different experimental conditions, different versions of the task, demographic characteristics, etc. Nevertheless, as will be shown, the theory which is obtained from this procedure can be represented in a stringent way and can certainly measure up to the rigor and consistency of otherwise common theories. So, in this section, we condense the introspective-empirical results to a comprehensive model in order to draw appropriate conclusions for mental agency in counting in the next step. According to the analytical dimensions presented in the (sub-) categories of (B), (C), and (D), theory building proceeds from general process features over counting strategies to FA/OM-dynamics. While the first and the last of these steps are not specific to counting, the second step explicitly includes this topic. In this way, counting is embedded in more general contexts of cognitive processing which are primarily accessible to first-person observation and thus also point to a consciousness-centered theory of counting.

**Fig. 2:**
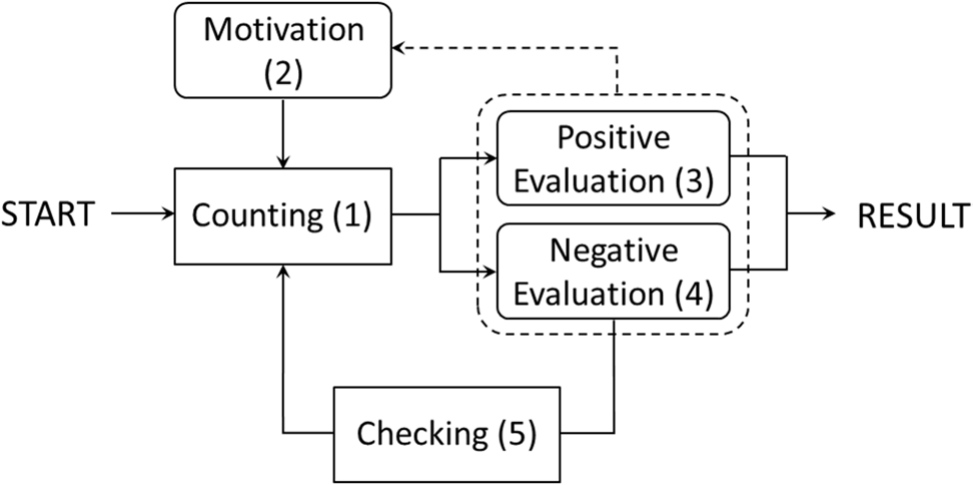
General process dynamics model for B-categories. The different forms of text boxes refer to mental actions (squared) and mental states (rounded), as explained below (see Sect. 4)

Starting with the dimension of general process features (B), the question is how the categories can be brought into a consistent context. First, beyond the designation, a processual interpretation is suggested by the logical relationship between the categories: A counting attempt leads to a preliminary result which is evaluated by the subject according to its adequateness. Depending on whether this assessment is positive or negative, there are different consequences. If negative, the subject will probably be inclined to check his or her result by a second count. In case of a positive evaluation, one has only to wait for the correct number to be announced. Moreover, evaluation regulates the motivation of the subject which is influential for subsequent counting attempts. Second, as an empirical argument indicated above (Sect. 2.3), difficulty in assigning text-fragments to categories can also be advantageous in theoretical regard if intermediate positions between the categories occur. These can be understood as links or mediating transitions between the categories which in turn can be interpreted as diachronic process phases. While above examples were given for inter-categorical positions between B1 (counting) and B2 (motivation), and between B1 and B3/B4 (positive/negative evaluation), here is an example for B2 and B3: “For me it was motivating for the next sequence if I had the right number of points in the sequence before” (Part. 2). In sum, we propose an integration of the B-categories in terms of a general process dynamics model (Fig. 2). Since in this model the counting-phase could be replaced by other kinds of problem-solving, it represents a generalized problem-solving cycle (Bransford and Stein 1993). While many approaches elaborate this on the respective content-level of problems, available knowledge, and strategy selection (Davidson and Sternberg 2003; Prezenski et al. 2017) others conceive problem-solving in close connection to self-regulation (Zimmerman and Campillo 2003; Perels et al. 2005). In our model, both perspectives are present as the process is driven by two feedback loops one of which refers to content-related evaluation of the (interim) result and, if necessary, repetition of counting whereas the other takes the self-related (motivational) impact of success or failure into account. However, in contrast to existing models of problem-solving, we would like to point out that our model does not result from third-person methodology, but is based entirely on first-person data. Although it may appear similar to common algorithmic or computational models, it is not about reducing the underlying first-person experience and mental activity to symbolic or quantitative parameters (which, for example, would be required for computer simulation), but rather to clarify the pre-reflective process structure which obviously can be made conscious. This is not to discredit the conventional approaches, but rather to highlight the potential convergence of results from both methodologies. In the following, this path will be pursued further by developing refined theoretical aspects according to the subdimensions of the counting strategies (C)—representing the content-level—and of FA/OM-dynamics (D)—referring to the micro-gestures of mental activity as overarching counting and self-regulation.

**Fig. 3:**
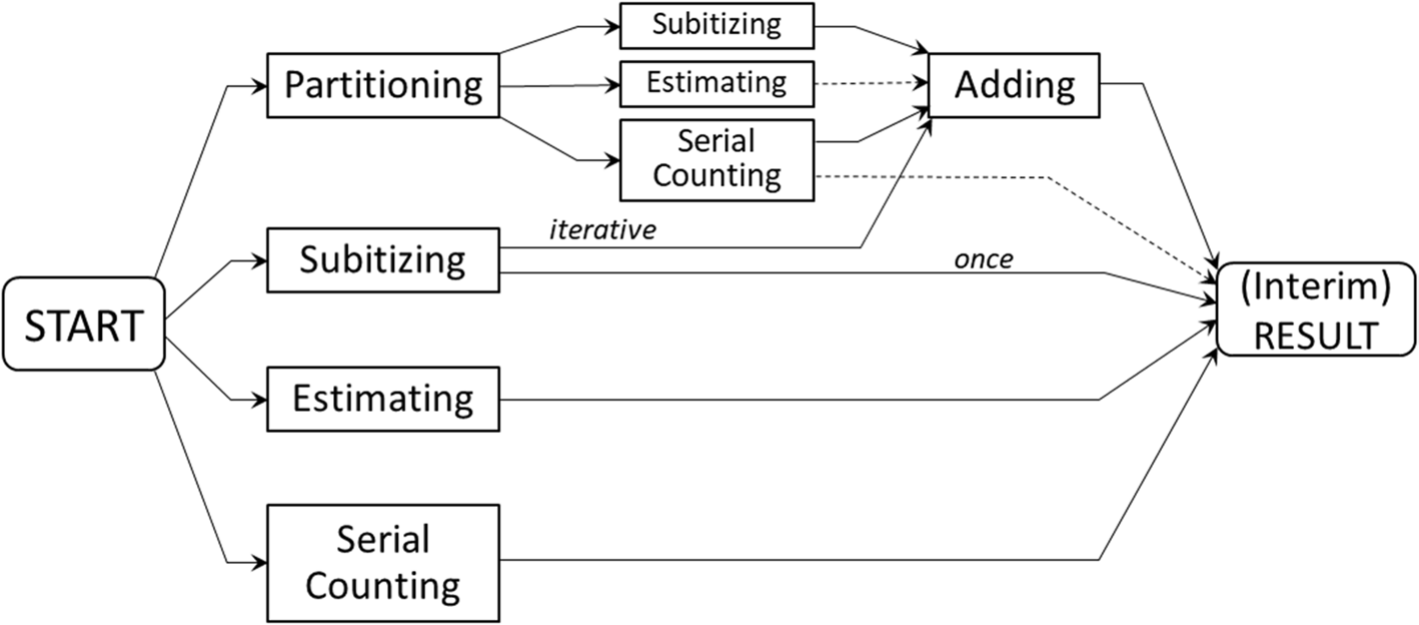
Counting strategies. Strategies and combinations reported in the first-person data are summarized in this scheme illustrating C-Categories as a refinement of Category B1 (Counting). The solid arrow-lines are explicitly confirmed by data, whereas the dashed lines represent connections that could not be extracted from the data, although they appear necessary (partial estimating) or, at least, possible (oriented serial counting)

The theoretical integration of the counting strategies (C) brings together their specific features with the diversity of their application (Table 3). While the quantitative analysis confirms the deployment of each strategy by at least half of the subjects (Table 4) and the usage of multiple strategies by most of them (Table 5), the individual forms of use cannot be derived from this. However, a comparison of text-fragments in Tables 1 and 3 (e.g., Parts. 8 and 9) suggests that counting strategies were applied in combination rather than isolated. Insight not only into the combination of strategies, but also into their situation-adaptive use and the development of their selection over the trials requires recourse to the complete protocols. Although this represents a further step in qualitative data analysis, it is dealt with in this section because it allows immediate modeling. In Table 7, we show combinations of the three known quantification principles with both auxiliary techniques of partitioning (a) and adding (b) as well as data-excerpts representing adaptive selection, combination and development (c). A schematic overview of all combinations extracted from the data can be seen in Fig. 3, where two not explicitly occurring (necessary or possible) paths are included. While the data in Table 7 substantiate this processual scheme as a further refinement of Category B1 (Counting), some aspects with theoretical relevance go beyond that. Particularly interesting is the diversity of individual strategy selection and combination, which would not have been revealed without introspective observation. From a problem-solving perspective, our model can be understood as an adaptive tool-box from which the subject heuristically chooses a strategy according to external conditions (e.g., number and speed of dots) and internal concerns (e.g., accuracy, effort) (Newell and Simon 1972; Payne and Bettman 2001; Todd et al. 2005). Whether the external environment or the deciding individual is more important in strategy selection is mostly answered in favor of the former, not least in order to prevent “the homunculus problem of needing a meta-heuristic to select the appropriate ‘tool for the job’” (Newell 2005, p. 12)”. However, since in our model this problem can be alternatively solved by counting on potentially self-conscious agents who can report about their strategy selection, no speculative homunculus is needed. It should be noted that this does not suspend the significance of the environment, but rather balances it in relation to mental agency.

**Table 7:**
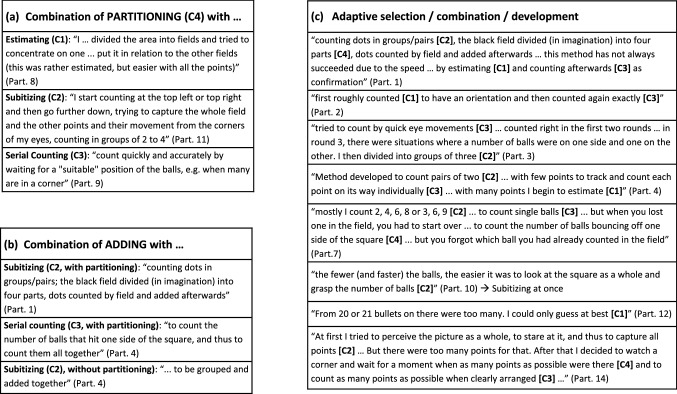
Counting strategies: combination, adaptive selection and development

Theory building regarding D-categories can also be contextualized first through certain aspects of problem-solving before arriving at an integrative approach to the quantification principles. Having the data coded according to FA/OM-dynamics in mind (Table 6), there is promising evidence of two different kinds of attentional processing in problem-solving which are mostly associated with analytical/convergent and creative/divergent thinking (Guilford 1956; Stanovich 1999; for overview see: Sowden et al. 2015). In the broader context of real world problem-solving, however, there is nothing to be said against extending the dual-process model to perceptual processes, especially since the shift between both forms of attention is assumed to be facilitated or triggered by intervening events in which the external environment is also involved (Sarathy 2018). Current neurophysiological studies concretize such events in mental impasse or failure to which analytical or convergent solving attempts can lead and which give rise to switch to creative or divergent forms of cognitive processing (Sprugnoli et al. 2017). The complementary mental activities associated with this are characterized as focused attention (FA) and defocused attention (DA) the former of which is seen as consciously controlled, while the latter is believed to be unconscious and automatic in nature (Kaufman 2011; Sowden et al. 2015). This view, however, can be challenged and extended by our results at several points. First, while our data coded according to focused attention fit well with the planful and convergent features of analytical processing, there is a conceptual difference between defocused attention and open monitoring. In contrast to a state with phenomenally negative connotation (DA), two different aspects can be extracted from the data coded according to OM. On the one hand, temporary departures from commitment to the task can be found (“looked out of the window”, Part. 14) while, on the other, a holistic attitude towards the stimulus is adopted and the reference to the task explicitly maintained (“capture the whole field and the other points and their movement from the corners of my eyes”, Part. 11). We interpret this as varying degrees of mental activity turning away from stimulus-related manifestations and, at the same time, opening for broader conceptual contexts as well as for an evaluation and, if necessary, change of strategy. Hence, DA reflects only one of these OM-aspects while the other is neglected.

Our second critique of the FA/DA-dual-process paradigm challenges the dictum of an unconscious and automatic characteristic of the DA-, or better, OM-Phase. Apparently, the test persons observe at least part of this phase and report on success, failure, and strategy change, too. Moreover, in the context of meditation, both FA and OM are considered as processes that can be voluntarily evoked and consciously experienced (Lutz et al. 2008; Slagter et al. 2011). Strikingly, there are clear parallels between the mental processes involved in FA-/OM-meditation and conventional notions on attention such as, for example, the monitoring and controlling of the attentional focus (Posner and Petersen 1990). In addition, training effects by meditation are often obtained via successive phases of FA- and OM-meditation which is consistent with the above-mentioned shift from analytical to creative thinking. However, since OM-meditation requires an enhanced monitoring faculty, it cannot be equaled with unconscious defocused attention, but rather includes “an increasing emphasis on cultivating a ‘reflexive’ awareness that grants one greater access to the rich features of each experience, such as the degree of phenomenal intensity, the emotional tone, and the active cognitive schema” (Slagter et al. 2011, p. 4). Third, as indicated in the quote, OM also allows to monitor “the active cognitive schema” what here is consistent with both evaluation and change of strategy. Connected with reflexive awareness of “the emotional tone” which does not only refer to the content-side but also to the activity-side of the experience the stage of evaluation (B3/4) passes over into motivation (B2, see Fig. 2). Exactly because subjects have metacognitive feelings about their mental performance (Proust 2013), they are responsible for adapting the strategy, which can be understood as emotional self-regulation in problem-solving (see above). Regarding mental agency, one can also speak of self-efficacy (Bandura and Adams 1977; Wagemann 2020), which is partly dependent on task-related success, but partly also builds independently on one’s own attempts experienced as coherent (see below).

**Fig. 4:**
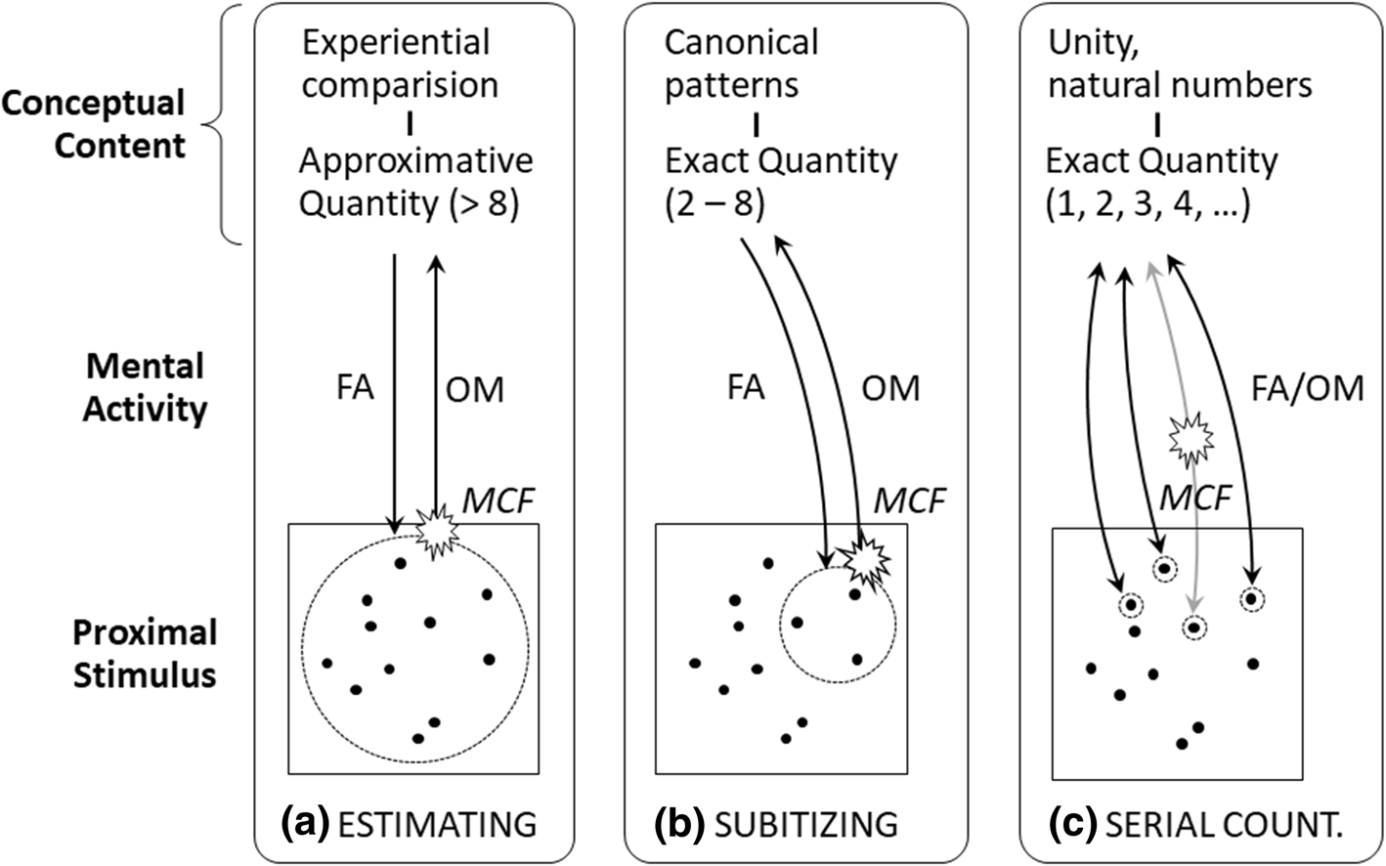
Mental dynamics in quantification. *FA *focused attention, *OM* open monitoring, *MCF* metacognitive feeling (positive/negative)

Now an integrative and consciousness-immanent model of the three counting principles shall be presented, which at the same time can be understood as a generalized approach to problem-solving (Fig. 4). As an alternative to approaches relying on competing hypothetical subsystems (ANS, OTS) and their neural correlates, we propose to explain estimating, subitizing, and serial counting as variations of the mental basic structure of perception as introduced by Rudolf Steiner and Herbert Witzenmann (Steiner 2011; Witzenmann 1983). While certainly not contradicting behavioral and neurophysiological findings, the mental basic structure is inherently driven by mental activity and thus operates on three phenomenal levels. Equally for all counting principles, it is assumed that a conscious result is achieved by unifying certain parts of the perceptual field (proximal stimulus) with suitable conceptual content conveyed by mental FA/OM-activity. This mental activity habitually works at a pre-reflective level but can be brought to consciousness under appropriate conditions (e.g., introspective tasks, meditation, see also Reyes and Sackur 2018). Also, in Buddhist meditation “FA and OM styles can be seen simply as two sides of the same coin” which underlines the integration of both forms of activity (West 2016, p. 232). In estimation, the numerical concept is not as sharp as in the other cases; it may include a certain range dependent both on the total quantity of objects and on individual experience. For subitizing, speed and accuracy are supported by pattern recognition, which means that smaller numerical concepts are directly linked to regular geometrical figures. In serial counting, the essential numerical concept is the unit (one), since each object to be counted must be recorded individually. Here, in contrast to the other two principles, mental effort directly depends on the total quantity. While with estimating and subitizing, it is only one sequence of FA/OM-activity leading to a result, serial counting requires mental tracking and enumeration of each object enabled by individual FA/OM-sequences.

This model also explains the difference in evaluation (as indicated in Fig. 4), because in serial counting it does not refer to a single number as a possible result, but to whether one has correctly grasped and counted all objects. In this context, the above statement of Participant 8 that she was able to distinguish already counted and uncounted items despite their uniform appearance and uncoordinated movement, points to evaluative metacognitive feelings (MCF) as indicators. In estimating and subitizing, however, such feelings indicate the experienced quality of fit between a numerical concept, introduced by FA-activity, and the stimulus. In case of a poor fit, subsequent OM-activity may lead to a repeated attempt with a different concept or even to a change in strategy. If the fit is experienced as consistent, OM-activity prepares the introduction of a next concept to another segment of the stimulus—or relaxes briefly before continuing the task. With serial counting, evaluation works quite similarly, just with the difference that MCFs do not refer to the (stimulus-related) turning point between FA and OM within one sequence but enable monitoring of the completeness of several FA/OM-sequences. While this modification demarcates serial counting from estimating and subitizing, it nevertheless allows for an explanation of adaptive strategy change during one trial. Since each FA/OM-shift includes the option for concept change—i.e., the “divergent” or “creative” part of the process—a series of several FA/OM-sequences cannot only run with “one” as number concept (serial counting) but can also apply different number concepts according to estimating and subitizing. Such generalized “serial counting” with variable (fuzzier or sharper) number concepts (> 1) or even concepts referring to a metacognitive “options context” also explains the option of strategy change (Baars 1988, p. 303). Furthermore, the necessity of adding partial results stemming from both repetition or combination of strategy illustrates the conceptual proximity of adding to counting. For example, one could estimate the number of dots in the lower part of the screen (first FA/OM-sequence with partitioning/estimation), immediately recognize a pattern of three dots in the upper part (second FA/OM-sequence with subitizing), count the rest one-by-one (third to last FA/OM-sequence with serial counting) and finally add it all up (see Table 7). Surprisingly, in this model serial counting is not only one of several counting principles, but also the prototype of their adaptive combination and perhaps even the mechanism underlying them (Cheyette and Piantadosi 2019). Again, this shows that the various counting principles are more closely interrelated from a phenomenal first-person perspective than would be expected in the context of most conventional approaches.

That it is nevertheless possible to draw insightful connections to established theories has already been shown and will now be briefly extended for the latter point with view to the influential Workspace/Working Memory Theory. As alluded in the previous paragraph, Baars’ (1988) approach equips consciousness, at least partly, with a metacognitive access to different options to be anticipated and chosen in the context of an ambiguous hierarchy of goals. Of particular interest to us is his idea of defining this place of action (the *global workspace*) as a mental area in which information relevant to controlling behavior becomes conscious through directed attention. Moreover, the comparison and prioritization of conflicting goals pertaining to different hierarchical levels can be interpreted as an attentional dynamic which comprises narrower aspects (e.g., individual items to be counted, as a lower level) and wider ones (e.g., the current counting strategy to be evaluated and perhaps changed, as a higher level). This seems to be supported by Cowan’s notion of “hierarchical shifting of attention” by which, shifting forth and back between the levels, the accomplishment of complex tasks is enabled (Cowan 2001, p. 93). Another aspect to be mentioned is the role of contextual coherence ascribed by Cowan to the *chunks of information* which are formed by a flexible conceptual web integrating isolated stimuli into meaningful unities. According to the situation and the capacity of the subject, chunks can be limited to individual stimulus items but can also include more complex structures. While there seems to be a capacity limit of four chunks which can be held in short term memory, the possibility to form groups of chunks (and even “supergroups” of groups of chunks) may explain subitizing as a fast and additive combination of such groups. When taking both aspects of attention dynamics and conceptual integration/differentiation together, one finds a certain closeness of the Global Workspace account and our structure-phenomenological interpretation of the data. Then situation-adaptive forming and processing of chunks appears to be equivalent to the basic structure of consciousness or, more specific, to the FA/OM dynamic performed in serial counting with flexible concepts ranging from unity over other numbers up to counting strategies. Again, this emphasizes the possibility of a detailed convergence of first-person research with theories developed under behaviorist or cognitivist paradigms, though this should not blind us to the different methodological and ontological perspectives from which they are derived.

## Discussion

A comprehensive discussion of the proposed account considering all methodological, psychological, and philosophical aspects would certainly exceed the scope of this study. Hence, we will limit ourselves to relatively brief comments on these points culminating in the philosophical perspective that has so far only been present in the background. Initially, despite our efforts to explain the interdisciplinary methodology used here, we are aware of possible criticism in this regard. The claim to combine different, partly even opposing research traditions certainly runs the risk of not doing justice to any of them. In retrospect, however, we believe that the atypical combination of methodological elements can be justified by the rich results and consistency of the theoretical model. Besides specific features of the model that will be addressed below, its status as a testable hypothesis is safeguarded by the experimental design with reproducible conditions, independent non-expert participants, non-reactive data acquisition, and some quantitative analyses. This necessary step towards third-person behavioral research is balanced by the nature of first-person data and its predominantly qualitative analysis from the perspective of mental activity. According to the three subordinated coding levels, different dimensions of this core topic were elaborated which can be treated as independent hypotheses about general process features, counting strategies, and mental dynamics but which also interlock across the categories. For analysis and modeling in the context of FA/OM-dynamics, the data basis is admittedly thin compared to the other categories. However, it is understandable that laypersons without systematic introspective or meditative training and explicit information about mental activity forms tend to focus on the content-related aspects of the task. It is therefore remarkable that only few, rather fragmentary, but quite clear and differentiated statements on FA and OM were made by as many as two thirds of the participants. With trained subjects perhaps more data could have been expected in this regard. But knowing what to look for in introspective observation can of course be discredited as implicit prejudice or conceptual bias (Danziger 1980; Schwitzgebel 2016) or associated with the observer-expectancy effect. However, this might as well be recognized as an appropriate refinement of the conceptual web necessary to capture any observation content in the sense of inevitable theory-ladenness (Popper 1959; Hanson 1958; Bogen 2016). Since the mental processes to which observation-guiding concepts such as FA and OM refer are pre-reflective, i.e., distant from untrained awareness, they may appear speculative from this perspective. Nevertheless, with additional support by the attention and problem-solving literature, this does not call into question the accessibility of these processes through conceptual guidance of introspective observation. In order to make this access as comprehensible as possible, we decided to make the gradual refinement of the conceptual framework transparent in data analysis, instead of leaving this in the more implicit form of data acquisition by trained subjects.

The integrative model of counting principles is also in favor of this methodology. The fact that all aspects of counting described in the literature (and with partitioning even beyond) can be consistently explained in a consciousness-immanent form suggests a complementary approach to these processes, which no longer need to be considered as necessarily subpersonal and to be reduced to computational models and neural mechanisms. In the context of the Approximate Number System (ANS), for instance, reference is still made to the accumulator model to derive mental representation of discrete natural numbers from accumulation of analog energy portions in a storage (Meck and Church 1983; Gallistel and Gelman 2000; Norton and Alibali 2019). This model, obviously inspired by computer hardware components (e.g., analog-to-digital converter, arithmetic logic unit), however, falls short to explain normalization of real number measures to unitized integers as well as numerical order (Butterworth 2016; Ulrich and Norton 2019). Since no neurophysiological equivalent of the postulated “pulse former” could be proven so far, intended to be “inserted into the stream of impulses, so that for each count there was a discrete increment in the contents of the accumulator” (Gallistel and Gelman 2005, p. 565), another explanation by our model can be envisioned. Instead of attributing computational principles to mental processing—which already presuppose what should be explained—discrete unity is always experienced by the subject when single or grouped objects are conceptually grasped as such and individuated at the stimulus by focused attention. As mentioned above, it is precisely the unlimited variety of concepts actualized by open monitoring which define and demarcate countable sets of any heterogeneous, volatile or also non-physical entities. Therefore, it seems more appropriate to denote the potentially self-aware originator of mental FA/OM-activity as “pulse former” than a speculative neural construct.

From the perspective of the Object Tracking System (OTS) as the other standard model of counting, the question of how perceptual stimuli integrate into countable sets defined by certain features is answered by a pre-attentive indexing and tracking mechanism (Pylyshyn and Storm 1988). This model, which is discrete from the outset in contrast to the ANS, relies on internal but at the same time distally anchored reference pointers (so-called FINSTs) and their temporal maintenance, even if the stimulus changes or disappears over time. However, it seems inconsistent to claim that the features intended to provide binding of incoherent (e.g., heterogeneous, ambiguous, cross-modal) stimuli are already present on the visual display or the retina (Pylyshyn 2001, p. 180/181). While there certainly is an early, non-conceptual stage of perceptual processing, any grasping of separated objects requires elementary conceptual reference as becomes clear with demonstratives (“this” or “that”) pointing to “something” (Steiner 2011; Witzenmann 1983; O’Shaughnessy 2000). Similar as in the short note on theory of science above, such non-propositional, rather gaze-directing references promise to arrive at point-like correspondences of their rudimentary, yet conceptual content with parts of the stimulus and, moreover, are only effective in connection with additional (e.g., spatial or temporal) determinations which are also conceptual in nature. In sum, features as a defining basis of reference pointers cannot be abstracted bottom-up from sensory stimuli. According to our model and in addition to the above critique of ANS, it is plausible to assume conceptual coherence at all descriptive levels as emerging independently of sensory data uptake and rather as actual or habitualized productions of mental activity. This is underpinned by the same functional role of elementary numerical concepts and adaptive counting strategies in our model both of which serve as means of problem solving in engagement with the perceptual field (while working at different levels). Moreover, this view is compatible to a certain extent with the top-down assumptions of “an innate grasp of concepts” and “processing abilities for combining and applying [..] representations” (Rips et al. 2008, p. 638) or of “an innate logical hypothesis space” (Barner 2017, p. 565). Whereas Rips’ ideas have been criticized for being logo-centric (VanMarle 2015), we emphasize that our approach does not build on implicit formal knowledge of natural number axioms, but rather rests on pre-formal universal regularities which are actualized and individuated by mental activity in many different forms of expression (Weger and Wagemann 2015b; Wagemann et al. 2018).

Having underlined the significance of mental activity and conceptuality as first-person aspects of counting which are mostly neglected in standard approaches we now come to philosophical implications for mental agency. This will tackle the question of whether there is not only relevant introspective access to mental activity in counting procedures, but to what extent it can also be attributed an agentive status. In general, it can be said that an individual performs a mental action when using one or more of their cognitive abilities and thereby achieving a certain effect. Different criteria for mental agency depend on what relation is assumed between the initiating cognitive capacity and the effected outcome. When this relation is considered as distant, indirect and particularly without the option of further control, the scope of mental phenomena deserving to be called mental actions appears to be rather restricted (Strawson 2003; Mele 2009). Mental actions are then limited to triggering cognitive processing which hereinafter remains withdrawn from monitoring and controlling access what might be construed as a concession to subpersonal automaticity in the sense of computational and neuro-centric approaches. Contrary to this view, at least two aspects of more direct participation are conceivable even after the first initiation of the cognitive process both of which are related to phenomenal consciousness in a certain way. Some philosophers consider the possibility that mental behavior can take the form of trying to achieve a certain goal to be a suitable criterion for mental agency (Proust 2001; Peacocke 2007). In analogy to physical action, others emphasize the crucial importance of conscious intentions which span the temporal extension of exercised mental activity in order to qualify it as mental action (O’Shaughnessy 2000). A further issue for an assessment of mental action is whether its outcome is limited to the delivery of propositional content or if it can also have other, so to say content-free effects. If the first case was true, as argued by Strawson (2003), the inconsistency between the necessity of already having a content in order to perform an intended action and the fact that it cannot be present before acting, would undermine the agentive status of mental activity. Although mental agency is mostly associated with content delivery, there is evidence of top-down mental activity in meditation leading to procedural or content-free awareness (Wagemann 2011b; Upton and Brent 2018; Winter et al. 2020). Beyond meditation, experience of non-conceptual content in perception makes clear that mental content brought about by intended mental acts does not necessarily have a propositional form (O’Shaughnessy 2000; Pylyshyn 2009; Wagemann 2018).

Initially, at least in our case, Strawson’s “content paradox” can be solved with view on the empirical part of this study. When starting the task, the participants have the clear intention to count the dots, but of course not yet a result. Therefore, the initial intention might include the range of possible results (numbers between 6 and 23) but not what can only be achieved by one’s own counting activity. That this activity contributes in such a way to the production of the counting result that it can be qualified as mental action is now to be justified by a closer look at the empirical results and theoretical modeling. Here, the insight is crucial that the result does not “ballistically” penetrate the participants’ consciousness but is produced by them in a stepwise controlled and reflected way. This is demonstrated first by the diverse, individually prioritized and combined counting strategies intentionally used for situational adaptation what enables the process to be conducted in close contact (Tables 3, 4, 5, 7, Fig. 3). Secondly, in the broader context of problem solving, explicit monitoring was found not only of the counting process, but also of its integration into the general process dynamics, which goes beyond counting in the narrower sense (Tables 1, 2, Fig. 2). According to the functional role and performative character of the five process characteristics (B-categories) in the double feedback loop, we propose to differentiate them into mental actions and mental states standing in dynamic relation to each other. In addition to Category 1 (counting) also the data encoded in Category 5 (checking) allow to ascribe these coordinating activities an agentive status because they do not appear to be automatically forced but rather individually intended and subsequently reflected in terms of their success or failure. Since the verification of one’s result can be considered an experimental attitude, this also represents the above-mentioned criterion of trying. Although trying can be stimulated by certain mental states such as general motivation or negative evaluation of a previous attempt, this does not mean that it is caused by them. Conversely, the data indicate that trying actively intended by the participants leads to rather passively experienced states of evaluation (Cat. 3 and 4) and motivation (Cat. 2)—wherefore the arrow lines in Fig. 2 have different meaning depending on whether they originate in an action (squared text box) or a state (rounded text box). In other words, while counting and checking can be associated with the “sense of agency,” evaluation and motivation seem to be experienced by the ‘sense of ownership’ (Gallagher 2012)—both, however, transferred here to the mental case.

A further aspect of trying as a mark of mental action can be found in Buckareff’s (2005) notion of “proximal intention” (P-intention) which, in the general context of action, must be distinguished from “distal intention” (Mele 1992; Pacherie 2008). While, in our case, the distal intention would be to arrive at a correct result of counting within the restricted time, the proximal intention is needed to *initiate and sustain* the process on the level of counting strategies, their adaptive deployment and evaluation. To what, other than mental activity itself, should then the term “proximal” refer? Analogous to the proximal stimulus in the context of sensory perception, mental activity here refers to its own maintaining, monitoring and adapting performance during trying which consequently can be denoted a “mental stimulus.” We are therefore dealing with a dual role of mental activity, which on the one hand—from the effect-side—appears as a stimulating object and on the other hand—from the agentive side—as a (potentially) consciously acting subject, which is consistent with the above distinction between mental states and actions. How close these two sides of the same coin can come together probably depends on how consciously mental activity is carried out and, in this way, also its effects become conscious as one’s own. To the same extent, mental activity, which can also occur completely pre-reflective, passes over into conscious mental action. On this account, mental action is not a matter of abstract inference but rather a phenomenal quality that increases on a scale beginning with mental states (as effects of pre-reflective mental activity) and progressing to ever more consciously executed mental activity (as intended mental action accompanied and completed with proximal intention).

As a further refinement of Buckareff’s (2005) work, we can integrate Pacherie’s (2008) notion of “motor intentions” (M-intentions) operating at the physical level of action and transfer it to the mental case. However, since we would not ascribe intentions to the neural level, we propose to introduce *executive intentions* (E-intentions) as a purely mental equivalent to M-intentions, serving to realize by FA/OM-dynamics what have to be done at a microlevel according to the currently selected counting strategy or its change. While at the level of P-intentions counting strategies are selected, monitored, and evaluated, through E-intentions the “upstream and downstream dynamics” (Pacherie 2008, p. 182) or the “hierarchical shifting” (Cowan 2001, p. 93) of attentional activity, oscillating between the sensorial and the conceptual level, are executed. As Upton and Brent (2018) point out, focused attention and open monitoring (among other meditation techniques) possess procedural aspects that can be performed and monitored without an intended result of content delivery. Hence, successful trying on the level of FA and OM (as E-intentions) is not restricted to content delivery but can also be related to one’s own mental and affective states. In other words, a mental activity that might fail regarding the distal, result-oriented intention can simultaneously be successful in a proximal or executive, self-referential context, which is also relevant, for example, for self-control during mind-wandering (Weger and Wagemann 2018). Both of the topics last mentioned—meditation and mind-wandering—seem to distract from the main focus of this study. However, the analysis and modeling of elementary mental activity forms undertaken here shows their relevance beyond the horizon of counting, not only for basic research, but also for therapeutic and educational fields of practice, for example, which can profit from this connection. By pointing out that these mental activities can reach an agentive status, new perspectives of person-relatedness and emancipation in healing and development can open up.

## Conclusion

Counting as a mental process does not have to be attributed to non-mental, computer-like processes that presumably take place in the brain. Conversely, it is perhaps more likely that computer processes will turn out to be materialized derivatives of initially pre-reflective mental activities and that the role of neural processing will be determined otherwise (Wagemann et al. 2018). To the extent that mental activities are consciously performed, i.e., metacognitively intended, attempted, reflectively sustained and evaluated, they can claim the status of mental actions. If a relatively simple introspective counting task with non-expert participants already leads to an extension of the scope of mental action, then it can be assumed that more sophisticated experiments will continue this path. For a safe progression of this interdisciplinary research, it seems important to first examine mental activity in quite mundane examples (as counting, for example); from there, however, implications for more “esoteric” topics may arise (e.g., self, meditation) or those with a wider philosophical scope (e.g., hard problem of mind and brain). This adumbrates a line of research that is worth pursuing, even if there are still strong dissenting voices: “First-person science of consciousness is a discipline with no methods, no data, no results, no future, no promise. It will remain a fantasy” (Dennett 2001). This study has provided clear evidence to the contrary. It has shown that cognitive science can extend its methods in just this direction—without slipping into fantasy—and that philosophy can thus benefit from empirical means that are not only borrowed from the mainstream. Possibly, philosophy will develop in a way that deviates from Dennett’s prophecies.

## Data Availability

The full datasets generated and analyzed during this study are available from the corresponding author on reasonable request.

## Addendum: Overview of coding categories

(A) First-person experience and mental activity/action in counting (core category).
(B) General Process Features
  1. Counting
  2. Motivation
  3. Positive Evaluation
  4. Negative Evaluation
  5. Checking
(C) Counting strategies
  1. Estimating
  2. Subitizing
  3. Serial Counting
  4. Partitioning
(D) FA/OM Dynamics
